# Dead but not forgotten: complexity of *Acropora palmata* colonies increases with greater composition of dead coral

**DOI:** 10.7717/peerj.16101

**Published:** 2023-10-11

**Authors:** Abigail Engleman, Kieran Cox, Sandra Brooke

**Affiliations:** 1 Department of Biological Science, Florida State University, Tallahassee, United States of America; 2 Coastal and Marine Laboratory, Florida State University, St. Teresa, FL, United States of America; 3 Marine Station, Smithsonian, Fort Pierce, FL, United States of America; 4 Biology Department, University of Victoria, Victoria, British Columbia, Canada; 5 Hakai Institute, Calvert Island, British Columbia, Canada

**Keywords:** Photogrammetry, Topography, Fractal dimension, Rugosity, Habitat complexity, Reef ecology, Structural complexity, 3D

## Abstract

Coral reefs are highly biodiverse ecosystems that have declined due to natural and anthropogenic stressors. Researchers often attribute reef ecological processes to corals’ complex structure, but effective conservation requires disentangling the contributions of coral versus reef structures. Many studies assessing the relationships between reef structure and ecological dynamics commonly use live coral as a proxy for reef complexity, disregarding the contribution of dead coral skeletons to reef habitat provision or other biogeochemical reef dynamics. This study aimed to assess the contribution of dead coral to reef complexity by examining structural variations in live and dead *Acropora palmata* colonies. We used photogrammetry to reconstruct digital elevation models (DEMs) and orthomosaics of the benthic region immediately surrounding 10 *A. palmata* colonies. These reconstructions were used to quantify structural metrics, including surface rugosity, fractal dimension, slope, planform curvature, and profile curvature, as a function of benthic composition (*i.e.*, live *A. palmata*, dead *A. palmata*, or non-*A. palmata* substrate). The results revealed that dead coral maintained more varied profile curvatures and higher fractal dimensions than live or non-coral substrate. Conversely, *A. palmata* colonies with a higher proportion of live coral displayed more uniform structure, with lower fractal dimensions and less variability in profile curvature measures. Other metrics showed no significant difference among substrate types. These findings provide novel insights into the structural differences between live and dead coral, and an alternative perspective on the mechanisms driving the observed structural complexity on reefs. Overall, our results highlight the overlooked potential contributions of dead coral to reef habitat provision, ecological processes, and other biogeochemical reef dynamics, and could have important implications for coral reef conservation.

## Introduction

Healthy coral reefs are among the most biodiverse and productive ecosystems on Earth. Reef ecosystem services are thought to be inextricably linked to Scleractinian corals’ structural characteristics and provision of physical habitat (Perry et al., 2013). By transforming the physical environment, corals directly and indirectly, influence nutrients, light availability, habitat space, and organisms within their system (*e.g.*, Kaniewska, Anthony & Hoegh-Guldberg, 2008; Hamylton et al., 2013). Widespread coral loss, driven by both acute and chronic anthropogenic stressors, therefore has significant consequences for reef architecture and the entire coral reef ecosystem (Alvarez-Filip et al., 2009; Graham & Nash, 2013). Pinpointing the drivers underlying reef biodiversity and ecosystem dynamics is vital for conserving and restoring coral reefs and mitigating further loss of ecosystem functions.

Recent advances in imaging and photogrammetric reconstruction have improved our ability to measure coral reefs’ structural complexities and glean new insights into the roles of biotic and abiotic structures in these vital ecosystems. To date, the role of abiotic reef structures has been overlooked in coral reef research, and researchers often use live coral cover as a proxy for three-dimensional (3D) reef complexity in experimental design, data collection, and interpretation (MacDonald, Tauati & Jones, 2018; Yadav, Alcoverro & Arthur, 2018; Cattano et al., 2020; Pombo-Ayora et al., 2020). This is likely because the presence of live coral is positively associated with 3D reef structure, and when live coral is lost, the reef architecture tends to flatten over large spatial and temporal scales (Alvarez-Filip et al., 2009). Coral reefs’ 3D complexity is also often associated with the abundance and diversity of niche space and habitat availability, meaning live coral and 3D complexity are similarly regarded as drivers of biodiversity on reefs (Morse et al., 1985; Dean & Connell, 1987; McCoy & Bell, 1991; Tokeshi & Arakaki, 2012). Some researchers suggest live coral influences fish distributions, while others believe reef structure is a more influential driver of fish communities (Pratchett et al., 2008). Although there is historical merit to using live coral as a proxy for reef structure, this approach conflates the abundance of live coral with the 3D complexity provided by both biotic and abiotic reef structures, potentially limiting insight into coral reef ecosystem dynamics. Studies that disentangle the roles of abiotic and biotic reef characteristics can reveal how reef communities respond to the loss of live coral cover *versus* the collapse of physical reef structure (Wilson et al., 2006; Wilson et al., 2008; Polunin et al., 2009; Agudo-Adriani et al., 2016; Wismer et al., 2019) Ultimately, separating the ecological importance of live coral from that of reef structures is foundational to understanding how ecosystem perturbations shape coral reef communities, ecological processes (Wilson et al., 2006; Pratchett et al., 2008), and resultant ecosystem services across spatial and temporal scales.

Our study builds upon previous efforts to decouple abiotic reef structure from biotic coral cover by assessing the relative contributions of both living corals and dead coral skeletons to reef topographical complexity. The primary focus of this study is to determine whether incorporating dead coral into the evaluation of topographical complexity (*i.e.,* assessing both abiotic and biotic contributions) yields more comprehensive information about the structure of coral reefs, and thereby provides a more holistic view of reef topography. Our study focuses on *Acropora palmata,* an important reef-building coral that forms large, branched colonies throughout the tropical Western Atlantic and Caribbean (Hoeksema & Cairns, 2023). Understanding the relative ecological importance of live and dead coral is an urgent priority while wild populations of this critically endangered species remain (Williams & Miller, 2012; Rodríguez-Martínez et al., 2014; Chen, Shertzer & Viehman, 2020; IUCN, 2022).

We used structure-from-motion (SfM) photogrammetry, an established tool in topographic mapping, to digitally reconstruct and assess the 3D properties of *A. palmata* colonies. Since first applied to marine ecosystems two decades ago (Bythell, Pan & Lee, 2001), SfM has gained traction in the field as a non-invasive and effective method for obtaining high-resolution reconstructions and measurements of coral reefs (Burns et al., 2015; Young et al., 2017; Magel et al., 2019; Fukunaga & Burns, 2020; Couch et al., 2021). SfM techniques have revolutionized our ability to precisely measure complex reef structures at a higher resolution than traditional methods, such as the ‘chain and tape’ method, which are limited by the physical properties of the equipment used (*e.g.*, chain link size) and provide a coarse descriptor of structural properties (Storlazzi et al., 2016). SfM techniques also allow for complexity to be measured at various spatial scales, providing novel links between ecology, morphology, and geological or physical processes on coral reefs (Leon et al., 2015; Storlazzi et al., 2016; González-Rivero et al., 2017; Aston et al., 2022).

Using SfM and structural analyses, we quantified surface rugosity, fractal dimension, slope, planform curvature, and profile curvature of live and dead *A. palmata* colonies. These structural metrics are commonly used and provide ecologically significant predictors of community diversity, species abundance, organismal biomass, larval recruitment dynamics, and food or nutrient cycling within the water column (Risk, 1972; Morse et al., 1985; Moore, Grayson & Ladson, 1991; Basillais, 1998; Kostylev et al., 2005), which all contribute to ecosystem dynamics. We used these metrics to explore the ecological implications of dead coral on reefs by testing the hypothesis that *A. palmata* colonies’ structural properties differ as a factor of live coral. Our study objective was to test the null hypothesis that the structure of live and dead coral colonies did not significantly differ.

## Materials & Methods

### Study site

**Figure 1:**
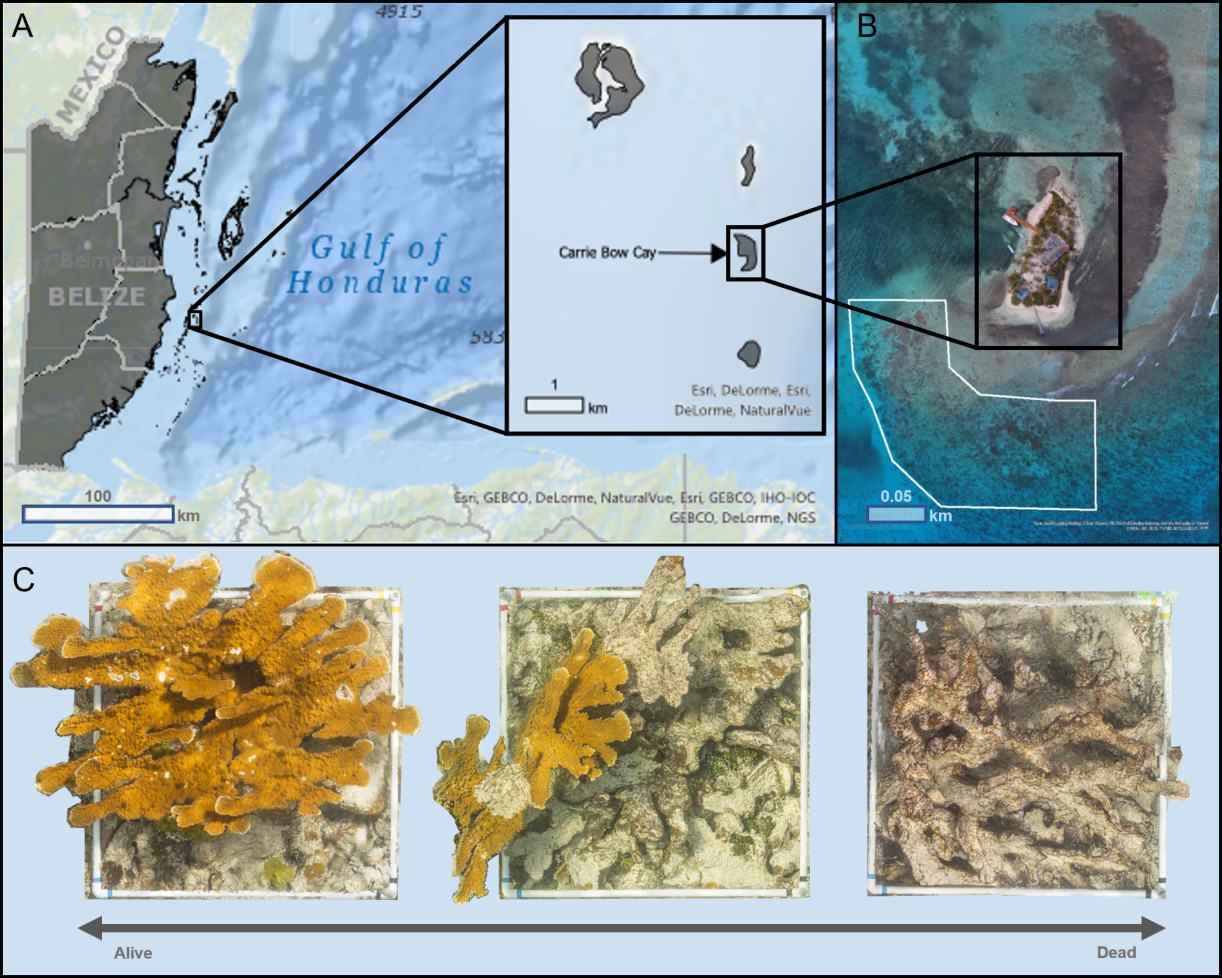
Map of Carrie Bow Cay, Belize (A) and the study site for structure-from-motion photogrammetric surveys (B) of *Acropora palmata* colonies (C). (A) Carrie Bow Cay (CBC), Belize, located along the Belize Barrier Reef. This map was built using ESRI ArcGIS^®^ World Ocean Base (basemap) and World Ocean Reference (Esri, Redlands, CA, USA), and a Belize-specific feature layer (created by the University of Edinburgh, adapted by Owen Lucas, 2018). Sources for World Ocean Base: Esri et al., 2020b; Sources for Ocean Reference: Esri et al., 2020a. All map images provided courtesy of Esri and are used herein with permission. Copyright/Source: Esri and its data contributors. (B) The study site for all *Acropora palmata* surveys was directly adjacent to CBC (shown in white). This visualization was created through Esri ArcGIS^®^ (Esri, Redlands, CA, USA) using an 8.89 cm/pixel orthomosaic created by Open Reef Mapping Society et al. (2018). (C) Acropora palmata colonies surveyed in this study ranged almost entirely alive (left) to completely dead (right). The three orthomosaics shown were generated as part of this study.

We conducted photogrammetric surveys on ten *Acropora palmata* colonies in September 2018 at the Carrie Bow Cay (CBC) Field Station, Belize, operated by the Smithsonian National Museum of Natural History (Fig. 1A). The colonies were located 50 m off the SW coast of CBC within a high-energy shallow reef (16.803, −88.08216), at depths between 1 m and 3 m (Fig. 1B). This reef had abundant *A. palmata* colonies that ranged from almost entirely alive to completely dead (Fig. 1C), providing sufficient target colonies for this study.

### Photogrammetric surveys

We visually assessed and selected colonies to capture a range of compositions from almost entirely alive to completely dead. We estimate that the selected *A. palmata* captured a range of 1% to 99% living colonies, based on our visual assessments (*e.g.*, color, surface texture and compositions, the presence or absence of living coral tissues; Fig. 1C). We also estimate that all dead corals have perished within the past five years. To control for the varying times since mortality among dead coral, we selected colonies of similar size (1–2 m^2^), with substantial vertical structure intact (*i.e.,* have not entirely degraded to rubble), without any visibly-noticeable macroalgal growths (which could bias structural assessments).

**Figure 2:**
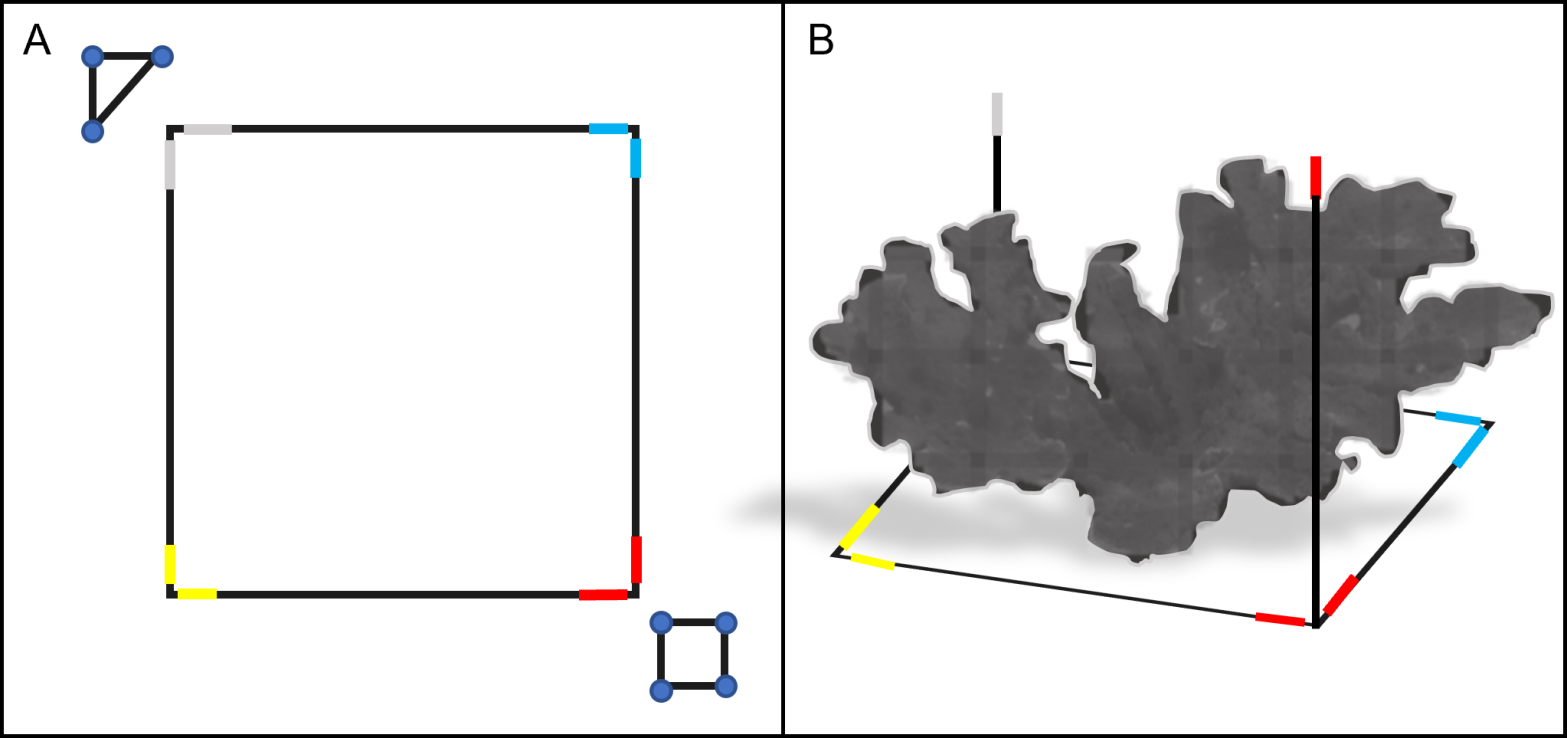
Schematic diagram of the PVC square and ground control points (GCP) used in the photogrammetric surveys. The 1 m × 1 m PVC square was placed around a target colony prior to survey (top-down view, A). The PVC square featured two 1 m vertical extensions on opposite sides (side view, B). Colored electrical tape was added to the corners and vertical extensions of the PVC square, to provide additional GCPs for reconstruction. A PVC triangle and a color board equipped with a 12-inch ruler were also used as GCPs for each survey (triangle and square shown in A). Together, these GCPs provided consistent reference points to improve model alignment, orientation and scaling during photogrammetric reconstruction.

We surveyed each colony using SfM photogrammetry techniques (Westoby et al., 2012), which involved placing a 3D 1 m × 1 m PVC square (with 1 m vertical extensions) and ground control points (GCPs) around the target colony before each survey to scale and refine model accuracy during post-processing (Fig. 2). We defined the survey region for each *A. palmata* ‘colony’ as all the area within the 1 m PVC square, and any attached colony structure that expanded beyond the 1 m square. The GCPs used in these surveys included a scale marker (triangle of known dimensions) and a color board. We also marked each side of the PVC square with a different color tape, which acted as additional GCPs and helped orient and scale the model.

**Figure 3:**
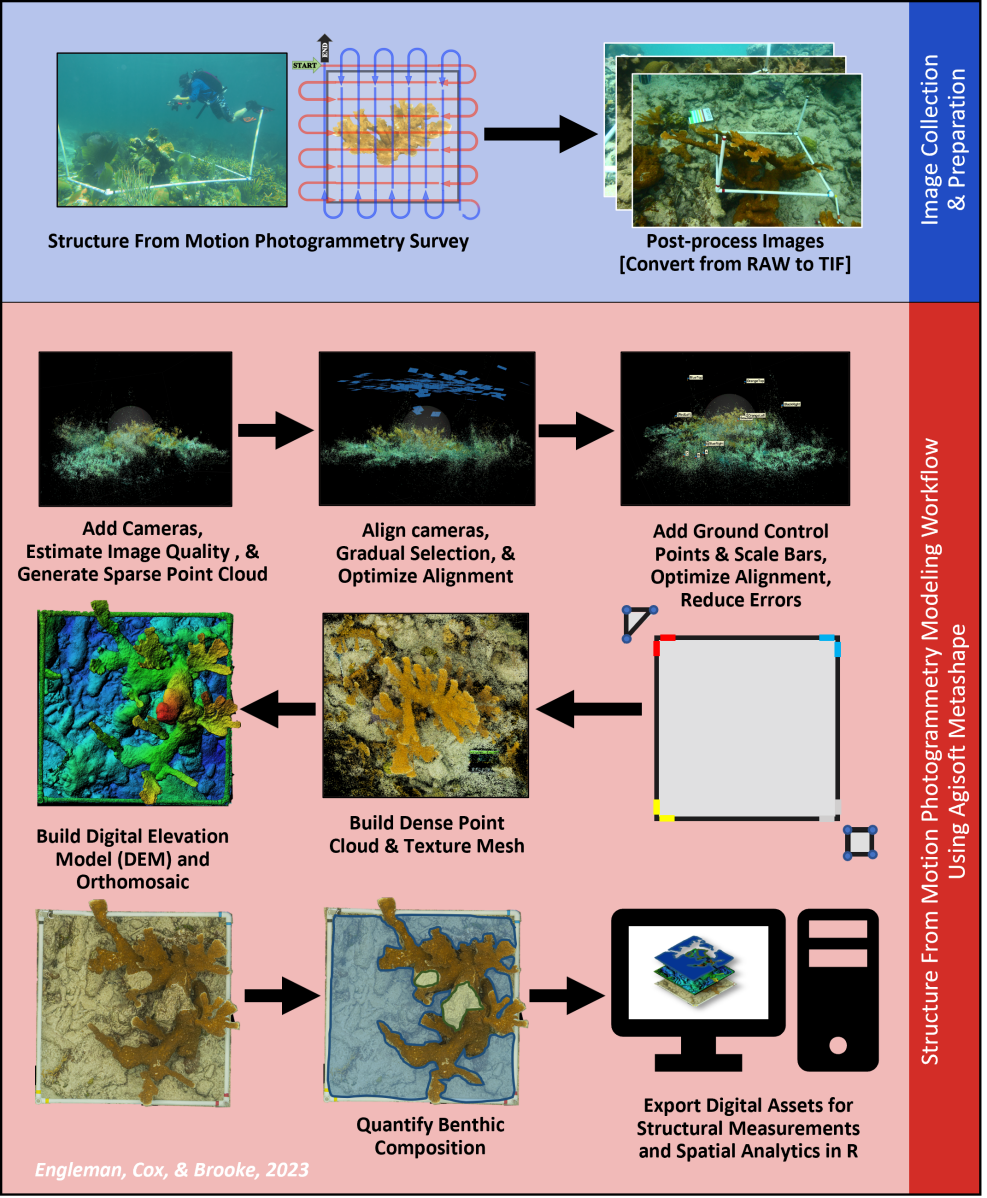
Structure from Motion (SfM) photogrammetric survey and modeling workflow. This workflow provides a visual representation of the steps involved in photogrammetric surveys and reconstruction. This diagram is meant to show the order in which the steps were executed. Note: Although each image was captured during this research, the images shown were selected to best convey a given step and therefore do not each feature the same *Acropora palmata* colony.

A Canon EOS Rebel T5, equipped with underwater housing and an attached dome port lens, took 18-megapixel CMOS (APS-C) RAW photographs of each *A. palmata* colony. Images were taken from above (perpendicular to the seafloor), starting at the scale marker, in a boustrophedonic pattern across the target colony (Fig. 3). Boustrophedonic refers to moving across the quadrat in straight lines, alternating the direction of each pass (*i.e.,* from left to right, then right to left, *etc*.), to reduce potential gaps in image coverage. Each photograph overlapped approximately 50–70% with the previous one, and over 300 photos of each *A. palmata* colony were taken. During each photogrammetric survey, a support diver noted the depth (in meters) at each corner and GCP. These values were used to ground-truth *z*-values during post-processing.

### Photogrammetric data processing

We used photogrammetric techniques to create a separate 3D model for each *A. palmata* coral colony. Post-processing and SfM workflows were carried out between September 2018 to July 2020 at the Smithsonian Marine Station in Fort Pierce, Florida, and Florida State University Department of Biological Science, using Agisoft Metashape (Professional Edition) software 2019 Version 1.5.5.9097 (Table S1). The SfM photogrammetry workflow used common points within overlapping images to construct a 3D model of each *A. palmata* colony survey region (Fig. 3). Our models were further scaled and oriented using the GCPs visible within our sampling images. We also used *in situ* depth values to ground-truth the models’ *z*-values, and orient colonies relative to their position within the water column.

**Figure 4:**
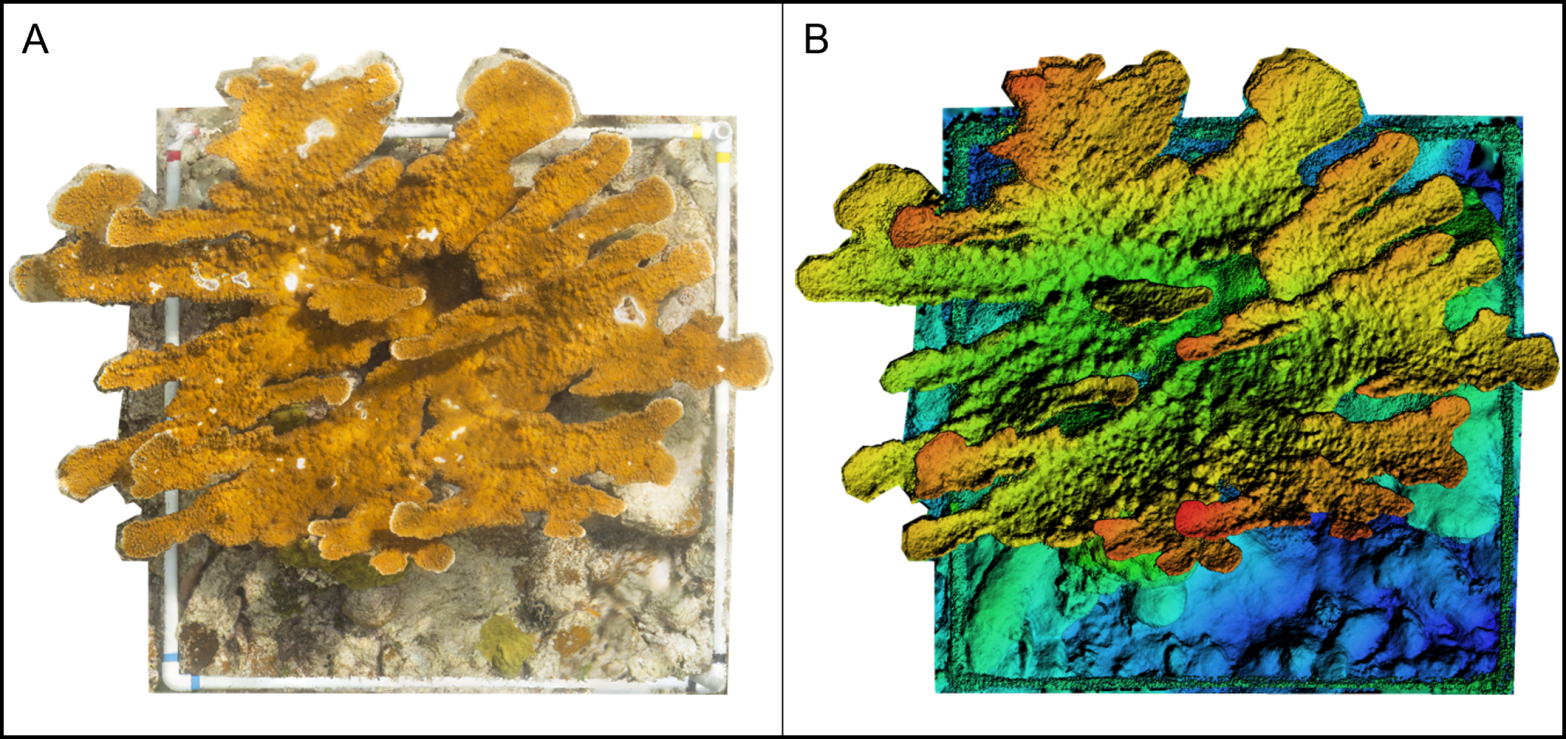
Example of an orthomosaic and a Digital Elevation Model (DEM) rendered in Agisoft Metashape for use in this experiment. (A) Spatially-rectified orthomosaic of an *Acropora palmata* colony area surveyed and reconstructed in this experiment. (B) A DEM constructed from the same *A. palmata* survey area.

We repeated the photogrammetric workflow for each survey, yielding 10 colony models. For each colony, the 3D model was used to produce two reconstructions: one DEM and one orthomosaic (Fig. 4). Each orthomosaic and DEM covered the full extent of the colony and comprised all benthic categories present within the colony survey region (see benthic composition, below). The orthomosaics were used for benthic categorization, and the DEMs were used to derive topographic metrics for each *A. palmata* colony.

### Benthic composition

We used internal Agisoft Metashape features to categorize benthic cover and quantify benthic composition on each orthomosaic. The orthomosaics allowed us to visually assess each colony and distinguish between regions of live coral, dead coral, or other benthos. The ’draw polygon’ tool was used to partition each orthomosaic into regions containing live *A. palmata*, dead *A. palmata* skeleton, or areas of benthos not associated with the *A. palmata* colony, termed “Live,” “Dead,” and ”Other Benthos,” respectively (and hereafter). We defined Live *A. palmata* as colony structures with living coral tissues, Dead as *A. palmata* structures without living tissues and Other Benthos as any non-coral substrate. While we carefully selected colony structures without (or with minimal) cover by other coral species, when present, we defined the non-*A. palmata* cover as Other Benthos.

We used the ‘Measure Polygon’ and ‘Generate Shape Report’ tools to quantify the area for each polygon by benthic category. Total Live, Dead, and Other Benthos area were quantified as the summative area for each category on the associated DEM (Fig. 3). Benthic composition for a given DEM comprised the total area of each of the three benthic categories.

### Structural complexity

Several topographic metrics were used to describe the structural complexity of each *A. palmata* colony, as described in Table S2. We quantified five metrics for each DEM analyzed in this experiment: surface rugosity, fractal dimension, slope, profile curvature, and planform curvature. Briefly, surface rugosity uses an area-based approach to characterize a surface’s 3D topography relative to its linear distance; fractal dimension is an index of a colony’s fractal geometry measured across an increasing step range; slope describes the elevation change over a specified distance; planform curvature quantifies the shape of a surface perpendicular to the slope; and profile curvature describes the rate of change in vertical slope at a given point (Table S2; Anelli et al., 2017; Yadav, Alcoverro & Arthur, 2018; de Smith, Goodchild & Longley, 2020). We calculated structural metrics at one cm^2^ spatial resolution in R statistical software version 3.6.1 (R Core Team, 2013), using the *raster* (Hijmans et al., 2020), *rgeos* (Bivand & Rundel, 2020), *sp* (Pebesma et al., 2020), and *ggplot2* (Wickham et al., 2020) packages, along with custom functions adapted from those created by (Fukunaga et al., 2019). We used the ‘aggregate’ function in the *raster* package to clip DEM raster edges based on a specified aggregation factor. Aggregation factor, *n*, generated *n* × *n* aggregated cells across the raster layer and replaced all aggregated cells located outside the DEM boundaries with a null value (Fukunaga et al., 2019). After removing null values from the layer, we converted the aggregated raster to a polygon and used the polygon to clip any non-aggregated cells from the original DEM. Although we established DEM boundaries before exporting each file from Metashape, this process applied consistent trimming specifications not available in Metashape, which were necessary to ensure subsequent metrics were calculated evenly across each replicate.

We calculated surface rugosity as the ratio of 3D geodesic surface area over the 2D ellipsoidal surface area (Jenness, 2004). We calculated the 3D geodesic surface area using the ’surfaceArea’ function, available in the *sp* package on R (Pebesma et al., 2020). Fractal dimension (*D*) was calculated as *D* = 2 - slope [log(S(*δ*)/log(*δ*)], based on DEM resolution ( *δ*), and 3D geodesic surface area for that resolution, S( *δ*), across spatial scales (Young et al., 2017). We quantified fractal dimensions across a step range from one cm^2^ to 32 cm^2^ (*i.e.,* at one cm^2^, two cm^2^, four cm^2^, eight cm^2^, 16 cm^2^, and 32 cm^2^ resolutions). To ensure that each *A. palmata* colony exhibited fractality (*i.e.,* demonstrated fractal properties across the observed step range - a prerequisite for quantifying *D*), we visually confirmed that there was a linear relationship between DEM resolution and surface area when plotted on a logarithmic scale. Once confirmed, the resultant slope of log-resolution *versus* log-surface areas was used to quantify *D* for each colony. As such, *D* values provided a measure of multiscale complexity, with values ranging from 2 to 3.

Slope and curvature were calculated for each raster cell, using the position of the cell relative to neighboring cells within a 3 × 3 window. We calculated slope, planform curvature, and profile curvature on each raster cell, then determined representative summary statistics (*i.e.,* mean, median, minimum value, maximum value, lower quartile, and upper quartile) for each of the three structural metrics. We used the interquartile range (IQR) to describe slope, profile curvature, and planform curvature in our analyses because when quantifying complexity at such small spatial scales (*i.e.,* at the colony-scale), mean values inadvertently oversimplify highly variable reef structures and conceal crucial structural information (Fukunaga et al., 2019; Pascoe et al., 2021).

### Statistical analysis

The benthic data collected in this study was compositional in nature and therefore analyzed using compositional data analysis (CoDA) methods. CoDA has rapidly evolved as a field of statistical theory that is capable of investigating entities of a whole, compositions of parts, and computations within the simplex (Bacon-Shone, 1992; Pawlowsky-Glahn & Egozcue, 2001; von Eynatten, Pawlowsky-Glahn & Egozcue, 2002; Aitchison, 2003b; Egozcue et al., 2003; Aitchison & Egozcue, 2005; Pawlowsky-Glahn & Buccianti, 2011; Pawlowsky-Glahn, Egozcue & Tolosana-Delgado, 2015; Filzmoser, Hron & Templ, 2018). CoDA methods overcome the complexities of compositional data geometries and the limitations associated with analyzing compositions using traditional multivariate techniques (Jackson, 1997; Pearson, 1897). Notably, CoDA methods overcome constant sum constraints that are characteristic of many analyses (*i.e.,* the assumption that all samples sum to a constant, such as 1 or 100%), enabling us to analyze DEMs of different total areas, in which compositional components could vary independently from one another (Aitchison, 2003a). Whereas the use of traditional statistical techniques on compositional data yields multicollinearity and unreliable estimates (Kucera & Malmgren, 1998; Aitchison, 2003b; Graham, 2003; Dormann et al., 2013; Douma & Weedon, 2019), advancements in CoDA methods provide valuable and broadly applicable techniques to assess compositional data, which continue to gain recognition across disciplines (Reyment, 1989; Baker, 2008; Campbell et al., 2009; Pierotti & Martín-Ferńandez, 2011; del Pozo Cruz et al., 2020). We performed all CoDA analyses in R, version 3.6.1 (R Core Team, 2013), using the *compositions*, *robCompositions*, and *zCompositions* packages (Palarea-Albaladejo & Martín-Fernández, 2020; Templ et al., 2020; van den Boogaart, Tolosana-delgado & Bren, 2020).

We treated all zeros within the compositional dataset as rounded zeros, meaning if any part of a component (*i.e.,* Live or Dead *A. palmata*) was present, it was below the detection limit (BDL) and, therefore, not detected during area quantifications. We defined detection limits as the ground sampling distance observed in each DEM. We treated rounded zeros using multiplicative substitution methods, which are sufficient when zeros comprise a relatively small portion of the dataset (Aitchison, 2003a; Martin-Fernandez, Barceló-Vidal & Pawlowsky-Glahn, 2003). This method calculated ’natural’ zero values to replace rounded zeros, allowing for log-ratio transformations with greater detection sensitivity between benthic cover (Aitchison et al., 2000; Martin-Fernandez, Barceló-Vidal & Pawlowsky-Glahn, 2003), at an alpha level of 0.05 for the determined detection limit (Palarea-Albaladejo & Martín-Fernández, 2020).

Compositional data with zero substitutions were log-ratio transformed within a closed simplex. We investigated the relationship between benthic composition and each structural habitat metric using robust linear regression, with the log-ratio transformed composition as the explanatory variables. Robust regression offered an approach tolerant to outliers and other deviations from model assumptions. This resulted in estimates for each composition component, which were fit using least trimmed squares regression (Aitchison, 1982; Hron, Templ & Filzmoser, 2010; van den Boogaart & Tolosana-Delgado, 2013; Filzmoser, Hron & Templ, 2018). The model outputs were displayed as predicted changes in the structure per increasing benthic cover by category.

## Results

### Digital elevation models (DEMs) and Benthic composition

Images of the *Acropora palmata* colonies yielded successfully aligned dense point cloud (DPC) models. Resultant 3D DPCs and DEMs achieved high resolution, with ground sampling distances (*i.e.,* resolution cm pixel^−1^) ranging from 0.057 cm pix^−1^ to 0.076 cm pix^−1^, between the 10 models (Table 1). The 1-cm^2^ DEM resolution used for structural metric calculations was substantiated by observed ground sampling distances below the exported models’ specified level of accuracy. Orthomosaic images, generated from overlaying orthorectified photos based on the DEM alignment, were exported alongside hill-shaded DEMs for visual comparison (Fig. 4).

Exported DEMs indicated that the surveyed benthic regions ranged from 1.20 m^2^ to 2.46 m^2^ (Table 1; Table S3). The mean planform surface area among the models was 1.44 m^2^ (± 0.40 SD). *Acropora palmata* colonies in the DEMs vary in depth from 0.104 m to 2.528 m below the surface, as determined by the minimum and maximum *z*-values between the DEM files.

Live *A. palmata* cover ranged from 0.17 m^2^ to 0.66 m^2^, with a mean cover of 0.50 m^2^ (± 0.18 SD) among all 10 colonies. Other Benthos covered an average area of 0.42 m^2^ (± 0.60 SD) among the 10 DEMs. Dead *A. palmata* cover was more prevalent than the other two benthic categories, with a mean cover of 0.52 m^2^ (± 0.42 SD) among all 10 DEMs. Other Benthos was composed predominantly of sand, although five DEMs contained small regions of other Scleractinian corals ($\bar {x}=0.01$ m^2^ ±0.009 SD).

### Structural complexity

We used spatial analyses to quantify surface rugosity, fractal dimension (*D*), slope, planform curvature, and profile curvature on each DEM (now referred to as ’raster’). Surface rugosity ranged from 2.64 and 4.79 between the 10 rasters, with mean surface rugosity equal to 3.60 (± 0.76 SD). Each *A. palmata* colony exhibited fractality over the one cm^2^ to 32 cm^2^ step range (*i.e.,* spatial scale). *D* averages across the observed one cm^2^ to 32 cm^2^ step range span from 2.28 to 2.46 with mean *D* equal to 2.36 (± 0.05 SD). Mean *D* associated with Live *A. palmata*, Dead *A. palmata*, and Other Benthos were 2.33 (± 0.006 SEM), 2.34 (± 0.006 SEM), and 2.36 (± 0.01 SEM), respectively.

**Table 1:** Digital elevation model (DEM) summary statistics.

	**Mean**	**SD**	**Median**	**Min**	**Max**
Ground sampling distance (resolution, m^2^ pixel^−1^)	0.00063	0.000062	0.0006	0.0006	0.00067
Planform surface area (m^2^)	1.444	0.396	1.327	1.202	2.459
Other Benthos (m^2^)	0.4228	0.5969	0.2757	0	1.8536652
Dead. *A. palmata* (m^2^)	0.5189	0.4178	0.5775	0.0002	1.044895
Live *A. palmata* (m^2^)	0.5025	0.1797	0.4745	0.1748	0.658649

**Notes.**

Ground Sampling Distance, the resolution (m2) per pixel achieved during the photogrammetric processing for DEM. export; Planform surface area –the 2-D surface area covered by each DEM.

Median slope values across the ten colonies ranged from 38.95 to 59.99. The average median slope was 48.45 (± 6.71 SD). Slope IQR varied from 37.74 to 47.96. Median planform curvature ranged from −2.76 to 0.92, with an average median value of −0.92 (± 1.12 SD) across all colonies. Planform curvature IQR varied from 46.42 to 141.37. Median profile curvatures among the colonies ranged from −0.72 to 9.02, with an average median value of 5.43 (± 2.93 SD). Profile curvature IQR varied between 83.56 and 272.53.

### Structural differences

**Figure 5:**
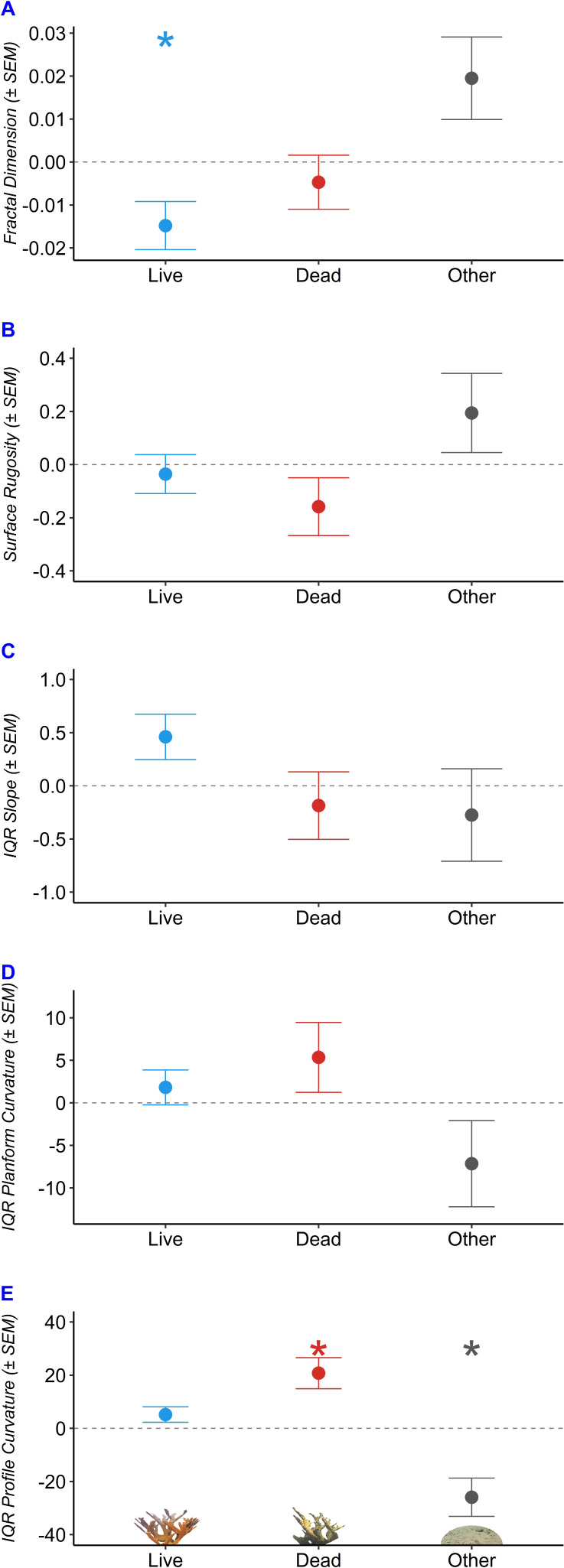
Structural characteristics of *Acropora palmata* colony surveys by benthic composition. Predicted change in structure with each 1 m^2^ increase in composition of Live Acropora palmata (“Live”), Dead A. palmata (“Dead”), and Other Benthos (“Other”), plotted for each of the five metrics: (A) Fractal Dimension, (B) Surface Rugosity, (C) IQR Slope, (D) IQR Planform Curvature, (E) IQR Profile Curvature. Asterix denote significance at the *a* = 0.05 level.

We used robust regressions to predict fractal dimension, surface rugosity, slope, planform curvature, and profile curvature as a function of benthic composition (Table 2, Fig. 5). Fractal dimension decreased by 0.015 per additional m^2^ of living *A. palmata* (*p* = 0.038), decreased by 0.005 per m^2^ of Dead *A. palmata* (*p* = 0.489), and increased by 0.019 per m^2^ of Other Benthos (*p* = 0.0896). Benthic compositions comprised of greater Live *A. palmata* were characterized by significantly lower *D* at the tested step range (one cm^2^ to 32 cm^2^; *p* = 0.0382). DEMs containing a greater composition of Other Benthos were positively associated with increased *D*, though it was not significant at the 0.05 *a* level (*p* = 0.090).

**Table 2:** Structural analysis predicts structural differences by colony composition. Coefficients and estimates from robust regressions executed to predict structural metrics based on the composition of Live *Acropora palmata*, Dead *A. palmata*, and Other Benthos.

	Benthos	$\Delta \hat {y}/{m}^{\mathbf{2}}$	**SEM**	*p*
*Fractal Dimension*	Live	−0.0148	0.0056	0.038^*^
	Dead	−0.0047	0.0063	0.489
	Other	0.0195	0.0096	0.090 .
*Surface Rugosity*	Live	−0.0358	0.0732	0.639
	Dead	−0.1584	0.1088	0.189
	Other	0.1942	0.1490	0.234
IQR Slope	Live	0.4604	0.2136	0.0681 .
	Dead	−0.1856	0.3174	0.5772
	Other	−0.2748	0.4346	0.5473
IQR Planform Curvature	Live	1.810	2.044	0.4163
	Dead	5.340	4.113	0.2508
	Other	−7.150	5.070	0.2175
IQR Profile Curvature	Live	5.171	2.905	0.1352
	Dead	20.745	5.844	0.0164^*^
	Other	−25.916	7.205	0.0156^*^

**Notes.**

$\Delta \hat {y}/m$^2^ –Predicted change in structural metric per additional square meter of alive, dead, or other benthos; SEM –standard error of the mean; p –*p* value; Significance levels of *a* = 0.05 and *a* = 0.10 are designated by the ”*” and ”.” Symbols, respectively.

Slope and profile curvature varied in response to changes in benthic composition. Slope (IQR) increased with an increasing composition of Live *A. palmata* (*p* = 0.0681) but was not affected by changes in Dead *A. palmata* or Other Benthos. The predicted IQR of profile curvatures was significantly influenced by the composition of Dead *A. palmata* and Other Benthos. Profile curvature IQRs were predicted to widen by 20.75 (± 5.844 SEM) with each additional m^2^ of Dead *A. palmata* (*p* = 0.0164). Profile curvature IQRs decreased by 25.916 (± 7.205 SEM) with each m^2^ of Other Benthos (*p* = 0.0156). Given that the predicted median profile curvature across all benthic compositions was 5.4 (*i.e.,* slightly concave surface, when profile curvature equal to zero denotes a flat slope), and all DEMs contained both negative and positive profile curvature values, Dead *A. palmata* structures exhibited greater variation ($\Delta \hat {y}/{m}^{2}$ = 152.52 ± 3.55 SD, *p* = 0.0164) of both convex and concave features than Live *A. palmata* ($\Delta \hat {y}/{m}^{2}$ = 136.95 ±2.91 SD, *p* = 0.1352) or Other Benthos ($\Delta \hat {y}/{m}^{2}$ = 105.86 ±  7.21 SD, *p* = 0.0156). Neither surface rugosity nor planform curvature was significantly influenced by changes in benthic composition at the 1-cm spatial resolution.

## Discussion

This study employed SfM photogrammetry techniques to digitally reconstruct DEMs and applied compositional data analyses to compare the contributions of living *versus* dead *A. palmata* to the colony-scale structure. Our findings suggest *A. palmata* structural complexity is related to the composition of live *versus* dead coral within the colony, supporting our experimental hypothesis. However, the specific nature of this relationship contradicts the widespread consensus surrounding reef dynamics, which associates live coral cover with greater structural complexity. Instead, our results show that, at the observed spatial scale, the significance of live coral to complexity varies depending on the metric used to quantify structure.

Our results indicated that the composition of live and dead *A. palmata* did not significantly influence surface rugosity, but our results should be interpreted with caution. We quantified surface rugosity for historical evaluation because the popularized chain-and-tape method (Risk, 1972) has made rugosity the de facto quantitative descriptor of coral reef structure. While a prior study found surface rugosity sufficiently captured structural change at 1-cm resolution following a severe hurricane (Pascoe et al., 2021), we found that in the absence of a known large-scale disturbance event, surface rugosity lacks the precision required to differentiate between more minute structural changes (*i.e.,* compositional difference between live and dead coral structures). Prior studies assessing small-scale surface rugosity substantiated the limitations associated with rugosity measures, finding that colony-scale surface rugosity becomes less precise and less accurate as coral morphological complexity increases (Figueira et al., 2015; Lavy et al., 2015; Bryson et al., 2017). *Acropora palmata*’s inherently complex and branching morphology likely subjected colony-scale surface rugosity measures in this study to both spatially- and morphologically- induced errors. Rugosity values also change in relation to variations in surface area and volumetric elements of a 3D surface (Ferrari et al., 2017), which differed between each colony we surveyed. These limitations associated with the rugosity metric (*i.e.,* restricted insight into model precision *vs.* accuracy) prevented us from deciphering whether the measurements observed in our study were indicative of structural similarities or the metric’s low precision and high margin of error. Ultimately, the metric’s documented poor performance at small spatial scales (*i.e.,* colony-scales) and among specific morphotypes, and its sensitivity to changes in the survey area, made it a crude and unreliable measure of structural complexity in this study. Future studies should exercise caution when using and interpreting rugosity, as this coarse metric could bias or obscure relationships between living coral, dead coral and 3D structure in relation to reef processes.

In contrast to rugosity, fractal dimension offered a more reliable assessment of structural complexity as a function of colony composition. Fractal dimension is well regarded as a valuable metric for capturing 3D variation in benthic surfaces (Zawada & Brock, 2009; Leon et al., 2015; Young et al., 2017; Fukunaga et al., 2020). Fractal dimension integrates complexity obtained across spatial scales and is a relatively error-free and orientationally-invariant metric that is also highly sensitive and capable of detecting small-scale morphological irregularities (Zahouani, Vargiolu & Loubet, 1998; Reichert et al., 2017; Fukunaga et al., 2019). Prior research found that fractal dimension values obtained across the one cm^2^ to 32 cm^2^ scale capture the morphological complexity of branching corals and explain more structural variability than surface rugosity or slope (Fukunaga & Burns, 2020), making fractal dimension a superior metric in our study across the same spatial range.

Our study found that live and dead *A. palmata* do not share the same fractal properties: colonies with a greater composition of live *A. palmata* had lower fractal dimensions than those with greater compositions of either other benthic substratum (*i.e.,* Dead *A. palmata* or Other Benthos). We observed that dead *A. palmata* structures maintained greater multiscale complexity than live *A. palmata* structures across the observed one cm^2^ to 32 cm^2^ spatial scale. Most understanding of reef communities relies on the assumption that live coral is associated with greater complexity, making these findings particularly non-intuitive. While the prevailing theory that living coral cover correlates positively with structural complexity is based on the need for live coral to form reef structures, it fails to consider the extent to which habitat variability, regardless of composition, may increase physical complexity. For example, a prior topographic assessment of mussel beds also found that beds characterized by the largest fractal dimension were not the ones with the greatest percent cover by live mussels (Commito & Rusignuolo, 2000). The beds with high percent mussel cover exhibited habitat homogeneity, because the densely packed mussels filled in available gaps, creating a relatively smooth topographic surface. Similarly, coral structures with a greater composition of live *A. palmata* polyps results in a lower fractal dimension compared to Dead *A. palmata*. This is likely due to the densely packed polyps creating topographic consistency across the colony surface, which is not observed in dead coral structures.

The distinct fractal dimension between colonies comprised of live *versus* dead *A. palmata* indicates that separate processes govern these coral structures. We hypothesize this is due to the ecological significance of live coral, which is absent from dead coral colonies. For example, live *A. palmata* can offset environmental variability (*e.g.*, through differential genetic expression; Hemond, Kaluziak & v, 2014), which enables a colony to protect itself against damage (*i.e.,* that can cause structural changes) and maintain a relatively homogeneous structure compared to dead coral. Once a coral dies, the underlying biogenic *A. palmata* structure loses the organism responsible for maintaining (relative) structural homogeneity, and the structures become increasingly vulnerable to external forces (*e.g.*, boring or endolithic organisms). Without the living coral organism protecting against opportunistic microorganisms and bioeroders, the remnant structures become subject to heterogeneous alterations, which can quickly reduce structural integrity and cause breakage (*e.g.*, Fordyce, Ainsworth & Leggat, 2020; Tribollet & Cuet, 2019), increasing structural heterogeneity.

Studies quantifying fractal dimensions at reef-wide spatial scales found reef areas occupied by dense dead *Acropora* structures had relatively higher fractal dimensions than areas occupied by live *Acropora* (Purkis, Riegl & Dodge, 2006; Zawada & Brock, 2009). Acropora colony mortality was also the dominant driver of complexity (Purkis, Riegl & Dodge, 2006; Zawada & Brock, 2009). The prior findings suggest our results may hold true beyond the scale examined in our study. It is important to note that this does not imply a direct fractal relationship between colony-scale and reef-wide structures or even that the same mechanisms drive the fractal patterns observed throughout. Instead, these congruent findings derived using a reliable structural metric highlight the mechanistic importance of dead coral to habitat complexity on coral reefs. Further research should build upon this research to explore the drivers of structural variation between live and dead *A. palmata* and assess whether separate biogeochemical processes differentially influence the structures pre-and-post coral mortality. This research avenue will reveal important insights into the mechanisms shaping coral colony’s states (pre-and-post-coral mortality) and the ambient biogeochemical conditions that may have significant implications on the subsequent ecological communities.

While fractal dimension was a valuable metric in this study, it only captures one element of habitat complexity. Comprehensive habitat description should include supplemental complexity measures (Tokeshi & Arakaki, 2012). Among the other complexity metrics we measured, slope and planform curvature did not differ as a function of benthic composition, but profile curvature was significantly influenced by the composition of Dead *A. palmata* and Other Benthos. As the relative composition of Other Benthos increased, profile curvature IQR significantly decreased, approaching zero. This can be explained by the relative homogeneity of sand, which predominantly comprised Other Benthos. In contrast, Dead *A. palmata* was associated with greater profile curvature IQR, which is indicative of highly variable surface topography (*i.e.,* distribution of concave and convex surface features). Colonies with a greater composition of Live *A. palmata* maintained a relatively narrow profile curvature IQR, indicating a more homogeneous surface topography at the one cm^2^ scale.

Our results build upon prior research that found positive curvature values were likely associated with small holes and ledge-like reef structures, suggesting reef surface topography observed at one cm^2^ resolution had a stronger influence on curvature values than the morphology of live coral (Fukunaga & Burns, 2020). These findings demonstrate that the composition of intra-colony mortality may drive surface topography on coral structures, as evidenced by the differences in profile curvature IQR. The distinct profile curvatures we observed between Live and Dead *A. palmata* also suggest that post-mortem structural variability may be driven by external factors (*e.g.*, endolithic microorganisms and boring organisms) heterogeneously degrading coral structures. Additional research is needed to understand the drivers of this structural variability between live and dead coral, and to discern the ecological implications of distinct forces acting upon living *versus* dead coral. Understanding these factors in the context of partial colony mortality will fill knowledge gaps surrounding reef ecosystem functionality and will help researchers anticipate changes in ecosystem dynamics associated with the loss of live coral on reefs.

In identifying structural distinctions between Live and Dead *A. palmata*, our findings further allude to distinct reef processes associated with the biotic and abiotic structures. For example, profile curvature regulates the acceleration and deceleration of flow over a surface, suggesting that hydrodynamics surrounding Dead *A. palmata* structures may greatly vary from that around Live *A. palmata*. Thus, habitat created by Dead *A. palmata* may experience increased microturbidity, a heterogeneous distribution of nutrients and sediments, and more inconsistent regulation of biogeochemical cycling or other ecosystem processes. In comparison, habitats dominated by Live *A. palmata*. Dead *A. palmata* structures may be subject to stronger and more heterogeneous erosion and deposition forces, which are governed by profile curvature. Over time, these forces may further shape the abiotic coral structure, continually altering its complexity as the structure degrades. As these forces and the processes they influence are beyond the scope of our study, further research should investigate the potential feedback loops driven by structure and the flow schemes facilitated by structural variations as mechanisms for further enhancing the complexity of remnant coral structures. This could provide a fruitful avenue for better understanding postmortem structural shifts and the associated ecosystem phase shifts or changes in ecosystem functions over time.

Fractal dimension is a descriptive complexity metric from which researchers can infer ecosystem dynamics (Sugihara & May, 1990). Significant shifts in the fractal dimension indicate shifts in the structure governing or generating processes within the system (Sugihara & May, 1990). Previous ecological applications of fractal analyses yielded new perspectives on conventional ecological concepts, sparking the reevaluation of previously held beliefs (Morse et al., 1985; Schmid, 1999; Borda-de Água, Hubbell & McAllister, 2002; Lennon, Kunin & Hartley, 2002; Marsh & Ewers, 2013; Yakimov et al., 2014). Using fractal dimension to quantify structure in our study revealed colonies with a greater composition of Dead *A. palmata* were significantly more complex than Live *A. palmata* colonies. We found that changing the composition of Dead *A. palmata* and Other Benthos signaled significant shifts in the fractal dimension, suggesting separate processes govern each of the benthic components tested (Sugihara & May, 1990; Schmid, 1999). Shifts in the fractal dimension indicate structure-governing processes likely transition in response to colony composition (Krummel et al., 1987). This is most clearly explained by coral mortality or composition of Dead *A. palmata* within the colony, and the resultant shift of processes that subsequently shape the colony’s remnant abiotic structure. Importantly, these shifts defined notable boundaries over which ecological inferences should not be extrapolated.

These findings are important because, once identified, such reef dynamics may explain a larger amount of ecological variability than that provided by live coral cover and the inextricable 3D structure live coral provides. Furthermore, the boundaries identified in our study stress the importance of further decoupling the presence of live coral from the complexity provided by abiotic reef structures. Future research on the distinct processes governing live and dead coral structures can unveil shifting biogeochemical conditions or new, informative parameters which researchers can use to assess ecosystem and community dynamics. Identifying structure-governing parameters specific to each coral life-stage (*i.e.,* live *vs.* dead) could also help limit extrapolation beyond the identified process-governing boundaries and prevent conflating live coral cover with structural complexity.

Structure-governing processes could be physical, such as erosion and flow, as previously mentioned, and biological, such as boring and excavation by other organisms. Live coral also drives structure-governing processes by maintaining homeostasis in response to environmental variations (Bruno & Edmunds, 1997; Hemond, Kaluziak & v, 2014; Kemp et al., 2015; Webber & Huppert, 2020). We postulate that a combination of physical, chemical, and biological dynamics governs coral structures following mortality. Contrastingly, abiotic structures lacking live coral to maintain homeostasis may be governed by allogenic engineers, such as bioturbators, which become the dominant biological forces shaping remnant skeletal structures through internal and external multi-directional dissolution and erosion processes.

While exploring the governing processes was beyond the scope of our study, the emergent questions warrant thorough investigation in further research. Identifying the distinct form-generating and process-governing forces driving live and dead coral structures will pinpoint factors underlying critical ecosystem functions. Advancing this objective requires that future studies also address this knowledge gap by developing a standard treatment of dead coral colonies and abiotic structures in benthic assessments. The ecological roles of biogenic coral skeletons following mortality are considerably understudied. This omission is likely because this physical state lies beyond the scope of many coral reef studies and due to the prioritization of research that necessitates live coral organisms. Many studies disregard dead coral altogether and instead report live coral as the sole benthic descriptor. This strategy fails to consider the distinct processes governing living *versus* dead coral structures, which may underlie ecological reef processing. Other studies combine dead coral structures into a nondescript abiotic benthic category, alongside sand or rubble, failing to capture postmortem skeletal structures that facilitate unique dynamic biogeochemical, biogeographic, and metabolomic processes. Only once we accurately characterize reef benthic composition and identify the processes governing live and dead coral structures, can we decouple the effects of abiotic and biotic reef forms in ecological dynamics. If successful, this will illuminate the extent and impact of changing ecological functions associated with live coral loss.

Environmental heterogeneity is a widely recognized driver of species diversity that spans taxa, biological communities, and biomes (Stein, Gerstner & Kreft, 2014). Structurally complex habitats support diverse communities by increasing niche space, creating refugia from adverse conditions, and providing a range of resources for taxa (Tews et al., 2004; Stein, Gerstner & Kreft, 2014). The importance of structural complexity for facilitating diverse communities is well documented within the study of coral reefs (Gratwicke & Speight, 2005; Graham & Nash, 2013; Cox & Bright, 2017; Cox, Woods & Reimchen, 2021). However, these insights are predominately based on the relationship between living coral and associated fish and invertebrate communities. Furthermore, examinations of the ecological implications of coral death commonly assess mass mortality events (*e.g.*, Hughes et al., 2017; Hughes et al., 2018), during which coral richness and structure decline rapidly. Here, we provide insight into the ecological value of including dead coral within examinations of coral reef dynamics. Consequently, if future assessments exclude dead coral, they may fail to capture additional components of reef complexity and the ecological mechanisms they support. Further linking 3D structures with ecological assessments can inform conservation management to enhance biodiversity and reef resilience (Simmons, DelWayne & Eggleston, 2022; Helder, Burns & Green, 2022; Curtis et al., 2023). The ecological insights gained from concurrently assessing biotic, abiotic, and 3D reef characteristics will likely increase over time as changing climatic conditions, local disturbances, and anthropogenic stressors continue to create a myriad of reef conditions (Anthony et al., 2008; Hughes et al., 2017; Claar et al., 2020).

## Conclusions

Our study utilizes fractal geometry and topographic measures to offer novel insights into reef dynamics, which contradict broad assumptions. By combining informative structural metrics with compositional data analysis, we were able to decouple the influence of live coral from that of abiotic reef structures. Our findings highlight the overlooked importance of dead coral and other abiotic reef structures in shaping colony structural complexity, and we postulate on the processes that drive or are driven by these observed distinctions. We must, however, acknowledge certain caveats associated with our methods. For example, we generated DEMs using images taken from a planar angle (*i.e.,* bird’s-eye view), which means structural complexity on the undersides of *Acropora palmata* structures was not accounted for. This exclusion could obscure complexity or slightly bias our results, but because it affected all DEMs equally, it likely did not significantly influence our findings.

Our empirical study and ecosystem predictions are based on models of ten *A. palmata* in one region at a single time point and cannot provide a comprehensive understanding of structural changes over time. The temporal limitations of our study may also discount the role of time-since-mortality on colony structure. Additionally, our observations were restricted to measurements from one species, and extrapolating our findings to multispecies reefs or other species and morphotypes may oversimplify the effects of complex inter- and intraspecies variations. Researchers should also exercise caution when interpreting our findings in relation to other uses of fractal dimension, as fractal geometry comprises a suite of algorithms and methods that differ in applications and results (*e.g.*, von Koch, 1904; Mandelbrot, 1982). Researchers should only employ fractal analyses following a thorough inspection of the methodological pitfalls and misinterpretations that can arise from these techniques (Halley et al., 2004). However, with proper use, fractal analyses can yield valuable insights into multiscale structures and the mechanisms driving structure-associated reef processes.

Ultimately, how we define and quantify coral structures significantly affects our understanding of reef ecosystem dynamics. To effectively conserve and restore reef ecosystems, we must identify the relationships between structure, biodiversity, and ecological communities. Fortunately, rapid advances in technology continue to bring unprecedented opportunities to address these knowledge gaps through enhanced computational power, increasingly higher-resolution earth observation data, real-time data integration workflows, and the ability to render and quantify 3D structures.

Meaningful ecosystem conservation requires meeting habitat destruction and haphazard reconstruction with urgent, science-based action. We must move beyond crude rugosity measures and proxies for habitat structure to embrace robust metrics that more accurately capture the structural intricacies of coral reefs. High-quality descriptions of reef structures can deepen our understanding of the characteristics that shape and are shaped by reef communities, which is critical for optimizing and accelerating reef resilience in the Anthropocene.

##  Supplemental Information

10.7717/peerj.16101/supp-1 Supplemental Information 1 Photogrammetry parameters used to render colony reconstructions in Agisoft

10.7717/peerj.16101/supp-2 Supplemental Information 2 Area covered by each photogrammetric survey (*n* = 10) ”Total” refers to the total area covered by a survey. “Alive” refers to the area of Alive *Acropora palmata* recorded within the total area for a given survey. “Dead” refers to the total area of dead *A. palmata* found within the given survey area. “Other” refers to the survey area comprised of other benthos (non-*Acropora palmata* substrate, such as sand). All areas were derived from an orthomosaic generated for each colony survey region.

10.7717/peerj.16101/supp-3 Supplemental Information 3 Description of the five structural metrics used to quantify colony structure

## Additional Information and Declarations

### Data Availability

The following information was supplied regarding data availability: The data is available at FigShare: Engleman; Cox, Kieran (2023). Raw Data.zip. figshare. Dataset. https://doi.org/10.6084/m9.figshare.21919410.v1.
