# Supplementary material for: Dead but not forgotten: complexity of Acropora palmata colonies increases with greater composition of dead coral
Source: PeerJ. 2023 Oct 11;11:e16101. doi: 10.7717/peerj.16101 (PMC10576496; doi:10.7717/peerj.16101)
Supplement: Supplemental Information 1 [file peerj-11-16101-s001.docx]

Table 1: Photogrammetry parameters used to render colony reconstructions in Agisoft.

| Process | Settings |
| --- | --- |
| Estimate Image Quality | Images with quality below 0.5 were removed (Agisoft-LLC, 2020) |
| Align Photos | Highest quality, generic preselection enabled, 240,000 key point limit, 0 tie point limit (limitless) |
| Gradual Selection | Points were removed based on 1 pixel reprojection error, 200 reconstruction uncertainty, and 20 projection accuracy thresholds |
| Build Dense Cloud | High quality, mild depth filtering, reuse depth maps disabled |
| Build Mesh | Arbitrary surface type, high face count, interpolation enabled, calculate vertex colors enabled |
| Build Texture | Adaptive orthophoto mapping mode, mosaic blending mode, texture size/count 4,096 pixels, hole filling enabled, 3 bands, uint16 colors |
