# Supplementary material for: Dead but not forgotten: complexity of Acropora palmata colonies increases with greater composition of dead coral
Source: PeerJ. 2023 Oct 11;11:e16101. doi: 10.7717/peerj.16101 (PMC10576496; doi:10.7717/peerj.16101)
Supplement: Supplemental Information 2 — ”Total” refers to the total area covered by a survey. “Alive” refers to the area of Alive Acropora palmata recorded within the total area for a given survey. “Dead” refers to the total area of dead A. palmata found within the given survey area. “Other” refers to the survey area comprised of other benthos (non-Acropora palmata substrate, such as sand). All areas were derived from an orthomosaic generated for each colony survey region. [file peerj-11-16101-s002.docx]

| ***Survey*** | ***Total***  ***(area, m^2^)*** | ***Alive***  ***(area, m^2^)*** | ***Dead***  ***(area, m^2^)*** | ***Other***  ***(area, m^2^)*** |
| --- | --- | --- | --- | --- |
| **A** | 1.5151 | 0 | 0.856451 | 0.658649 |
| **B** | 1.2144 | 0 | 0.687986 | 0.526414 |
| **C** | 1.5016 | 0.952113 | 0.016544 | 0.532943 |
| **D** | 1.4017 | 0.758391 | 0.046561 | 0.596748 |
| **E** | 1.2988 | 0.10399 | 1.02 | 0.17481 |
| **F** | 1.2408 | 0.35107 | 0.467052 | 0.422678 |
| **G** | 1.3558 | 0.7996317885 | 0.0002172115302 | 0.555951 |
| **H** | 1.2534 | 0.006105 | 1.044895 | 0.2024 |
| **I** | 1.2017 | 0.200237 | 0.8015453 | 0.1999177 |
| **J** | 2.4592 | 1.8536652 | 0.2480616 | 0.3574732 |

Table S2: Area covered by each photogrammetric survey (n = 10). The “Total” refers to the total area covered by a survey. “Alive” refers to the area of Alive *Acropora palmata* recorded within the total area for a given survey. “Dead” refers to the total area of dead *A. palmata* found within the given survey area. “Other” refers to the survey area comprised of other benthos (non-*Acropora palmata* substrate, such as sand). All areas were derived from an orthomosaic generated for each colony survey region.
