# Supplementary material for: Dead but not forgotten: complexity of Acropora palmata colonies increases with greater composition of dead coral
Source: PeerJ. 2023 Oct 11;11:e16101. doi: 10.7717/peerj.16101 (PMC10576496; doi:10.7717/peerj.16101)
Supplement: Supplemental Information 3 [file peerj-11-16101-s003.docx]

Table A1: Description of the five structural metrics used to quantify colony structure.

| Metric | Description |
| --- | --- |
| Surface Rugosity | Surface rugosity is a type of rugosity that measures the 3D characterization of a topographical surface relative to its linear distance. Surface rugosity was introduced as an alternative to the conventional transect-based method for estimating reef rugosity, which uses a chain-and-tape approach to measure the length of a thin chain draped across the reef over the linear distance of the measured reef (Risk, 1972). Although rugosity is a widely-used measure of reef complexity. many researchers criticize it as a crude estimate of 3D reef complexity and a poor descriptor of underlying reef structures (Zawada and Brock, 2009; Friedman et al. 2012; Harborne et al. 2012; Anelli et al. 2017). Surface rugosity takes an area-based approach to measuring 3D coral complexity by comparing the 3D geodesic surface area to the 2D ellipsoidal surface area (Jenness 2004), This study includes surface rugosity for historical comparison. |
| Fractal Dimension | Fractal dimension is a multiscale measure of complexity and self-similarity, which quantifies how the irregularity of a shape changes at different scales (i.e., across a given “step range”). A high fractal dimension value (closer to 3) indicates a structure is highly complex and irregular, with self-similarity, or similar complexity of features across different scales. Objects with higher fractal dimensions are considered to fill more 3D space. Lower fractal dimensions measures (closer to 2) indicate an object has less a more homogenous and less complex structure (think of it as being closer to 2D than 3D). Researchers use fractal dimension to quantify marine landscapes because the metric is highly sensitive to small-scale structural variations and orientationally invariant, yet relatively error-prone (Zahouani et al., 1998; Reichert et al. 2017, Fukunaga et al., 2019). Quantifying fractal dimension in the context of natural structures can also reveal patterns and underlying processes shaping ecological systems. For example, natural forms with distinct measures of fractal dimension can indicate the structures are governed by separate processes. As such, fractal analyses have offered new perspectives on conventional ecological concepts (e.g., Morse et al., 1985, Schmid 1999, Lennon et al. 2002, Marsh and Ewers, 2013). |
| Slope | Slope describes change in elevation elevation over a specified distance (de Smith et al. 2020). When measured from a raster, slope represents the gradient, or steepness, of a raster cell relative to its neighboring cells (Burrough & McDonnell 1998). Slope metrics provide a representative measure of variation in surface angle relative to the horizontal plane (Kemp, 2008; Friedman et al. 2012). |
| Planform Curvature | Planform curvature describes the shape of a surface perpendicular to the slope. Positive planform curvature values indicate a surface is horizontally convex within a given cell, meaning flow along this surface diverts outward from the center of the slope (Kimerling et al. 2016). Negative planform curvature values indicate the surface is horizontally concave, and flow will converge from the sides toward the center. A zero value for planform curvature indicates the surface is linear, with no horizontal curvature. |
| Profile Curvature | Profile curvature describes the rate of change in vertical slope at a given point (De Smith et al. 2020). Profile curvature runs parallel to the slope and affects the acceleration (*i.e.*, positive profile curvature) and deceleration (*i.e.*, negative profile curvature) of flow processes along a surface (Kimerling *et al.,* 2016). Profile curvature measures describe bathymetric concavity and convexity along the slope of reef structures. |
